# AI-guided ethnopharmacology for cardiovascular drug discovery: from biomedical data to experimental validation

**DOI:** 10.3389/fphar.2026.1962508

**Published:** 2026-09-21

**Authors:** Shun Yao, Xiaoxin Chen

**Affiliations:** 1 Cardiovascular Research Institute, University of California, San Francisco, CA, United States; 2 Department of Physiology, University of California, San Francisco, CA, United States; 3 Eli and Edythe Broad Center of Regeneration Medicine and Stem Cell Research & Bakar Aging Research Institute, University of California, San Francisco, CA, United States

**Keywords:** artificial intelligence, botanical drugs, cardiovascular disease, ethnopharmacology, foundation models, natural products, perturbation-response prediction, single-cell transcriptomics

## Abstract

Despite substantial advances in pharmacological therapy, cardiovascular disease remains the leading cause of mortality worldwide. Botanical drugs represent a rich source of structurally diverse bioactive metabolites, yet their translation into modern cardiovascular therapeutics remains limited by chemical complexity, incomplete mechanistic understanding, and challenges in experimental validation. Meanwhile, the rapid expansion of biomedical data resources and advances in artificial intelligence (AI) have created new opportunities to systematically investigate botanical drugs beyond empirical approaches. In this review, we summarize the major biomedical data resources supporting AI-guided ethnopharmacology, including phytochemical and ethnomedicinal databases, multiomics datasets, single-cell and spatial transcriptomics, perturbation-response resources, biological interaction networks, and clinical evidence. We then discuss the computational approaches used for molecular property prediction, drug-target interaction prediction, perturbation-response modeling, and foundation-model transfer learning, highlighting how these complementary strategies address distinct stages of pharmacological discovery. Building on these advances, we propose two complementary AI-guided discovery frameworks: a compound-first strategy that predicts the pharmacological activities of botanical drugs and a disease-first strategy that identifies natural products capable of reversing disease-associated molecular and cellular states. We further discuss emerging applications in cardiovascular diseases, emphasizing the integration of AI with single-cell atlases, disease-state prediction, mechanistic investigation, and experimental validation to prioritize candidate botanical therapeutics. Finally, we outline current challenges in data standardization, model robustness, interpretability, and prospective validation. We propose that AI-guided ethnopharmacology should be viewed as a hypothesis-generation framework that complements, rather than replaces, pharmacological experimentation. When supported by standardized botanical materials, high-quality multimodal data, rigorous biological validation, and prospective clinical evaluation, AI has the potential to accelerate cardiovascular botanical drug discovery and facilitate the translation of ethnomedicinal knowledge into modern precision therapeutics.

## 1 Introduction

Pharmacological discovery has evolved from empirical observations of medicinal plants to mechanism-based drug discovery and, more recently, to data-driven pharmacology. Early medical traditions identified therapeutic plants through repeated observation, and historical records such as those attributed to Shennong preserved knowledge that later inspired pharmacological investigation. The development of medicinal chemistry enabled the isolation and structural characterization of bioactive metabolites, while advances in molecular biology established target-based pharmacology by linking these molecules to specific receptors, enzymes, and signaling pathways. High-throughput screening and omics technologies subsequently expanded drug discovery by enabling systematic analysis of chemical libraries and treatment-induced molecular responses (Drews, 2000; Pereira and Williams, 2007; Eder et al., 2014). More recently, advances in artificial intelligence (AI) and large-scale biomedical data integration have shifted pharmacology toward predictive, data-driven discovery. Figure 1 illustrates these major transitions and highlights how advances in AI now complement traditional pharmacological workflows by integrating chemical, biological, and clinical data.

**FIGURE 1:**
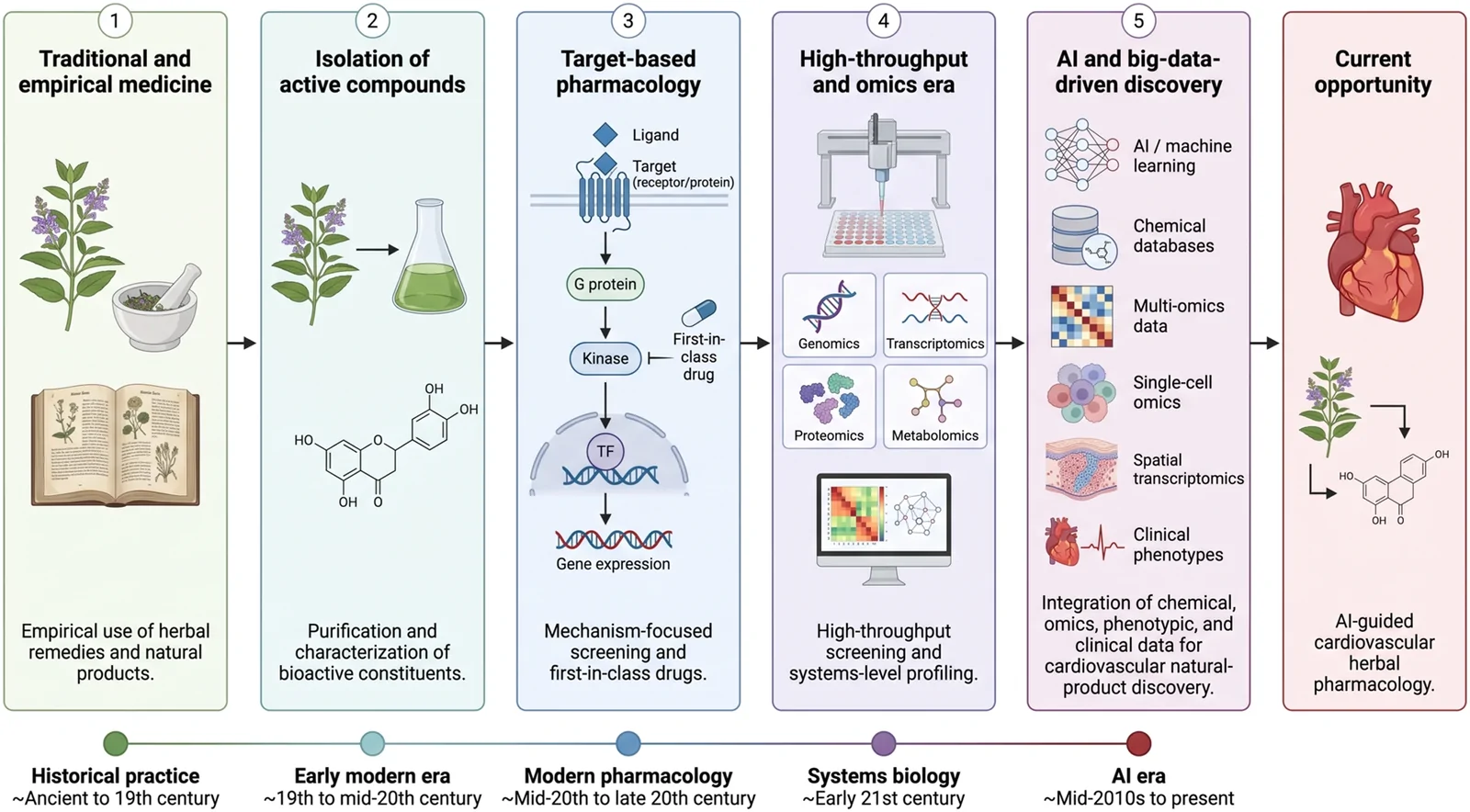
Evolution of pharmacological discovery from empirical medicine to AI-guided drug discovery. Pharmacological discovery has progressed from empirical use of medicinal plants to chemical isolation, target-based pharmacology, high-throughput screening, omics profiling, and AI-guided integration of biomedical data. AI complements rather than replaces experimental pharmacology by prioritizing candidate metabolites, targets, and disease mechanisms for experimental and clinical validation.

The rapid expansion of biomedical data has created a new challenge: pharmacological datasets are now too large and heterogeneous to be interpreted manually. Chemical databases contain millions of molecular structures, perturbational screens measure cellular responses to thousands of interventions, and clinical datasets capture diverse patient characteristics and outcomes. AI-driven computational methods can identify statistical relationships within these datasets and support tasks such as molecular property prediction, toxicity assessment, biomarker discovery, and candidate prioritization (Vamathevan et al., 2019). Their performance, however, depends critically on the quality, completeness, and relevance of the underlying data.

These challenges are particularly pronounced for botanical drugs and ethnopharmacology. A single botanical preparation may contain numerous metabolites, and its chemical composition can vary with plant species, source material, processing, and extraction methods. Traditional therapeutic uses often only partially overlap with modern disease classifications, while databases integrating medicinal plants, metabolites, molecular targets, symptoms, and diseases vary substantially in the strength of supporting evidence. Some associations are supported by chemical characterization or experimental validation, whereas others are inferred from literature curation or computational prediction. AI models that fail to distinguish among these evidence types may generate confident but poorly supported conclusions.

AI is therefore best viewed as a hypothesis-generation tool rather than a substitute for pharmacological experimentation. It can prioritize candidate metabolites, infer molecular targets and cellular responses, and identify potential safety concerns before labor-intensive experimental studies. However, model performance depends on rigorous evaluation, independent validation, and ultimately experimental confirmation. While previous reviews have surveyed AI across natural-product discovery broadly (Mullowney et al., 2023), this review focuses on the integration of cardiovascular disease context, cell-state and perturbation-response modeling, and experimental validation within complementary compound-first and disease-first workflows. We summarize the biomedical data resources and AI methods that support ethnopharmacological drug discovery, describe two complementary AI-guided discovery frameworks, and discuss cardiovascular applications, experimental validation strategies, and the remaining challenges for translating AI predictions into clinically useful botanical therapeutics.

## 2 Literature search strategy

This review was conducted as a structured narrative review rather than a systematic review; therefore, PRISMA-style study selection and exhaustive record counts were not applied. Relevant literature was identified through structured searches of PubMed, Web of Science, and Google Scholar for studies published through July 2026. Search terms included “artificial intelligence,” “machine learning,” “deep learning,” “foundation model,” “perturbation response,” “drug response prediction,” “drug–target affinity,” and “quantitative structure-activity relationship (QSAR),” combined with “ethnomedicinal plant,” “botanical drug,” “natural product,” and cardiovascular disease-related terms. We focused on primary studies describing computational methods for molecular property prediction, drug–target interaction, perturbation-response modeling, or transfer learning, together with their experimental or computational validation. Reference lists of relevant publications were also screened to identify additional studies. Priority was given to studies that clearly reported model inputs, computational methodology, evaluation strategy, and experimental validation. Official documents, including the Nagoya Protocol, the Kunming-Montreal Global Biodiversity Framework, and relevant CBD decisions on digital sequence information, were also consulted to inform the discussion of research ethics and benefit sharing.

## 3 Biomedical data resources

AI models do not learn pharmacology directly from experiments or nature. Instead, they learn from digital representations assembled from chemical databases, omics datasets, perturbation screens, biological networks, and clinical records. Together, these complementary resources connect botanical drugs with molecular mechanisms, cellular responses, and disease outcomes, providing the foundation for AI-guided pharmacological prediction.

### 3.1 Chemical and botanical databases

Chemical databases provide standardized representations of small molecules together with experimentally measured bioactivity. Resources such as PubChem, ChEMBL, BindingDB, and DrugBank link molecular structures with bioassay results, protein targets, pharmacological annotations, and clinical information (Kim et al., 2025; Zdrazil et al., 2024; Liu et al., 2025; Knox et al., 2024). These data support molecular property prediction, toxicity prediction, and drug–target interaction modeling by converting experimentally characterized metabolites into machine-readable representations.

Botanical drug knowledge bases complement conventional chemical databases by integrating traditional therapeutic uses with modern biomedical evidence. Resources such as TCMSP, SymMap, and HERB 2.0 connect medicinal plants with reported metabolites, molecular targets, disease associations, pharmacokinetic properties, and experimental or clinical evidence (Ru et al., 2014; Wu et al., 2019; Gao et al., 2025). These databases provide the initial knowledge layer for AI-guided ethnopharmacology by linking traditional medical knowledge with contemporary pharmacological data.

These resources should not be treated as uniform evidence. TCMSP, SymMap, and HERB combine experimentally measured, literature-curated, and computationally predicted relationships, while filters for oral bioavailability or drug-likeness may exclude relevant metabolites or propagate uncertain target assignments. NPASS provides quantitative activity and species-source records, but provenance and experimental context still require verification before model training or biological interpretation (Zhao et al., 2023). For each pharmacological claim, we propose an evidence hierarchy that distinguishes direct experimental support, indirect experimental or observational evidence, and computational predictions. The quality of the original study should be assessed by examining its methods, controls, biological relevance, and reproducibility. Publication or inclusion in a database does not by itself establish that a claim is valid. Traditional uses can guide candidate selection but do not establish molecular targets. Network and docking predictions should remain clearly labeled as hypotheses and should not be treated as experimentally confirmed data for model training or validation. Erroneous, unsupported, or pharmacologically implausible claims should be excluded from the verified evidence set, and the reasons should be recorded. Records derived from the same study should not be counted as independent evidence. Researchers should also check whether their conclusions hold when only the better-supported evidence is used.

A well-supported experimental claim illustrates how this hierarchy can be applied. Kong et al. (2004) showed that berberine increased LDL receptor expression in human hepatoma cells and that blocking ERK activation abolished this response. These results support regulation of LDL receptor expression in that model, but do not establish direct binding of berberine to the LDL receptor. A database entry based on this study should therefore describe an experimentally observed expression response rather than a confirmed binding interaction. The cell model, treatment concentration and duration, assay, and controls should remain linked to the entry (Kong et al., 2004).

A contrasting example is the predicted rutin–CYP3A4 interaction in a network pharmacology study of Rhododendron arboreum. The study reported a docking score of −11.0 kcal/mol and subsequent molecular dynamics simulations, but did not experimentally measure this interaction (Jangwan et al., 2024). An entry derived from these results should remain a low-confidence target hypothesis, not a measured binding affinity or confirmed therapeutic mechanism. It should not enter a verified compound–target training or test set without experimental support for the interaction. The absence of such evidence means that the prediction is unverified, not necessarily false.

### 3.2 Omics and perturbation resources

Omics and perturbation resources characterize how biological systems respond to genetic or pharmacological perturbations. Connectivity Map, L1000, and L1000CDS^2^ enable comparison between disease-associated and treatment-induced transcriptional signatures, facilitating disease-first drug discovery (Subramanian et al., 2017; Duan et al., 2016).

Single-cell and spatial datasets add information about where disease-related programs occur. The Human Heart Cell Atlas identified major cardiac cell populations and described their regional characteristics in the adult human heart (Litviňuková et al., 2020). Studies of myocardial infarction and cardiac tissue organization have further mapped transcriptional programs, chromatin accessibility, and cell–cell relationships across injured and non-injured regions (Kuppe et al., 2022; Kanemaru et al., 2023). These atlases can help determine whether a predicted target is expressed in the relevant cardiac cell type and whether a proposed molecular response aligns with a disease-associated cellular state.

### 3.3 Biological networks and clinical evidence

Network resources such as STRING, Reactome, KEGG, and Open Targets organize protein interactions, signaling pathways, and disease associations into interpretable biological frameworks (Szklarczyk et al., 2025; Milacic et al., 2024; Kanehisa et al., 2025; Buniello et al., 2025). These resources enable AI models to interpret predicted targets within their molecular and disease context.

Clinical records extend pharmacological research beyond controlled experimental settings. Electronic health records and administrative claims contain information about medication exposure, comorbidities, adverse events, and patient outcomes. The Observational Health Data Sciences and Informatics initiative and the OMOP common data model demonstrate how data from different healthcare systems can be standardized for reproducible observational analyses (Hripcsak et al., 2015). Real-world evidence is also used to guide drug-development decisions, monitor safety, compare treatment effectiveness, and explore potential new indications (Dagenais et al., 2022).

### 3.4 Integrating heterogeneous data

Taken together, these resources represent complementary layers of biomedical knowledge rather than independent databases. Chemical and botanical resources define candidate metabolites, omics and perturbation datasets describe biological responses, cell atlases identify disease-relevant cell populations, network resources provide mechanistic context, and clinical data connect molecular predictions with patient outcomes. Integrating these heterogeneous data enables AI models to move from botanical preparations to experimentally testable pharmacological hypotheses. These complementary biomedical resources provide the data foundation upon which AI models operate, whereas the computational methods used to analyze these data are discussed in the following section (Table 1).

**TABLE 1:** Comparison of computational methods in pharmacological prediction.

AI model class	Typical input	Primary output	Representative examples	Main strength	Key limitation
Molecular property/QSAR-like	Chemical descriptors, fingerprints, molecular structures	Activity, physicochemical property, or toxicity label	MoleculeNet benchmarks, DeepTox	Scalable screening of many compounds	Performance depends on representative labels and realistic splits
Drug-target affinity	Compound representation plus protein sequence or target features	Binding affinity or interaction score	DeepDTA	Produces a testable target hypothesis	Binding is not equivalent to cellular or clinical efficacy
Perturbation response	Baseline cell state, drug identity or structure, dose, covariates	Post-treatment expression or cell-state distribution	scGen, trVAE, CPA, chemCPA, CellOT, scVIDR, PRnet, PerturbNet	Models functional response and cellular heterogeneity	Requires expensive perturbation data; transfer can fail out of domain
Foundation-model transfer	Pretrained molecular or single-cell embeddings plus limited task data	Response in unseen or sparsely sampled contexts	CRISP NaFM, Geneformer	Potential to reduce task-specific data requirements	High computational cost and uncertain causal interpretability

## 4 Computational methods for pharmacological prediction

### 4.1 Representation of chemical and biological information

Before computational models can predict pharmacological responses, chemical structures and biological systems must be translated into numerical representations. Small molecules are commonly encoded using molecular descriptors, molecular fingerprints, SMILES strings, molecular graphs, or learned embeddings, each capturing complementary chemical information (Morgan, 1965; Weininger, 1988; Rogers and Hahn, 2010; Jaeger et al., 2018). These representations enable AI models to compare compounds, predict molecular properties, estimate drug–target interactions, and infer biological activity.

Biological systems are represented in a similar manner. Bulk RNA-seq provides transcriptome-wide descriptions of tissues or cell populations (Mortazavi et al., 2008), whereas single-cell RNA-seq resolves molecular heterogeneity across individual cells (Tang et al., 2009). Disease-associated and treatment-induced transcriptional signatures derived from these datasets can be compared with perturbation resources such as Connectivity Map and L1000 to identify compounds predicted to reverse disease-associated molecular states (Subramanian et al., 2017).

Single-cell transcriptomic data are inherently high dimensional and sparse. To facilitate prediction, deep-learning models commonly compress gene-expression profiles into low-dimensional latent representations (compressed numerical summaries of gene-expression patterns) that preserve cell identity and functional state while reducing noise. Variational autoencoders such as scVI and scGen are widely used for this purpose and form the basis for many perturbation-response models (Lopez et al., 2018; Lotfollahi et al., 2019).

Transcriptomic representations can be integrated with additional biological information, including protein sequences, dose, cell type, disease status, species, and experimental covariates. Encoding these variables enables AI models to predict how the same compound may behave across different biological contexts. For example, CPA explicitly separates basal cellular state from perturbation, dose, and experimental covariates, enabling prediction of responses in previously unseen cell types and treatment conditions (Lotfollahi et al., 2023).

Once chemical and biological information has been represented numerically, AI models can address distinct pharmacological prediction tasks. These methods can be broadly categorized into molecular property prediction, drug–target interaction prediction, perturbation-response modeling, and foundation-model transfer learning. Rather than competing approaches, they answer complementary biological questions and are therefore suited to different stages of drug discovery. Table 1 summarizes the representative inputs, outputs, strengths, and limitations of each model class.

### 4.2 Molecular property and drug-target prediction

Molecular property prediction represents one of the earliest and most widely used applications of AI in drug discovery. These models estimate physicochemical properties, biological activity, or toxicity directly from chemical representations and are primarily used for early-stage compound prioritization (Table 1). In ethnopharmacology, they provide a practical means of screening large collections of natural products before experimental evaluation.

Classical QSAR models relate chemical features to experimentally measured biological activities or molecular properties. Depending on the prediction task, regression models are commonly used for continuous outcomes such as IC50 or binding affinity, whereas classification models distinguish active from inactive compounds or toxic from non-toxic molecules. Early QSAR studies relied largely on linear regression, while later approaches adopted nonlinear algorithms, including support-vector machines and random forests, to better capture complex structure–activity relationships (Svetnik et al., 2003). Despite the rapid development of deep learning, these classical algorithms remain valuable because they perform well on moderate-sized datasets, are computationally efficient, and provide interpretable baselines for evaluating more complex models. Standardized benchmarks such as MoleculeNet further facilitate objective comparison of molecular property prediction algorithms across multiple pharmacological tasks (Wu et al., 2018).

Deep learning extends molecular property prediction by learning informative chemical representations directly from molecular structures rather than relying solely on manually engineered descriptors. DeepTox is a representative example, using multitask neural networks to predict toxicity across multiple Tox21 assays and demonstrating how shared representations can improve prediction of related pharmacological endpoints (Mayr et al., 2016). These approaches have substantially expanded the scope of AI-assisted molecular screening by enabling simultaneous prediction of multiple biological properties from a single chemical representation. Their performance can nevertheless deteriorate for natural-product scaffolds or assay conditions that differ substantially from the training data, so benchmark accuracy should not be assumed to transfer directly to new botanical metabolites.

A related application is drug-target interaction prediction, which seeks to identify proteins likely to interact with a compound. DeepDTA illustrates this strategy by jointly encoding compound SMILES strings and protein amino acid sequences to estimate binding affinity (Öztürk et al., 2018). Such models generate testable hypotheses for biochemical validation and can substantially reduce the number of candidate interactions requiring experimental screening. However, predicted molecular interactions should not be interpreted as evidence of therapeutic efficacy. Whether a compound ultimately modifies disease progression depends on additional factors, including tissue exposure, target expression, pharmacokinetics, downstream signaling, and disease context. Consequently, molecular property and drug-target prediction models are best viewed as tools for candidate prioritization rather than predictors of clinical efficacy.

### 4.3 Perturbation-response prediction

Perturbation-response models extend pharmacological prediction from molecular interactions to cellular responses by estimating how biological states change following chemical or genetic perturbation (Table 1). Unlike molecular property or drug–target models, which predict isolated chemical or molecular features, perturbation-response models aim to predict treatment-induced changes in gene expression, cell state, or cellular composition. This capability is particularly attractive for ethnopharmacology because botanical drugs often influence multiple pathways and cell types simultaneously, making cellular response a more informative endpoint than a single molecular target.

Many perturbation-response models are based on deep generative architectures that learn latent representations of cellular states before predicting their responses to treatment. scGen pioneered this approach by learning perturbation-induced transcriptional changes and transferring these responses across cell types, experimental datasets, and species (Lotfollahi et al., 2019). TrVAE subsequently extended this framework to unpaired biological datasets, enabling conditional prediction when matched control and treated samples are unavailable (Lotfollahi et al., 2020). CPA further improved perturbation prediction by explicitly separating basal cellular state from perturbation, dose, and experimental covariates (Lotfollahi et al., 2023). This design enables prediction across previously unseen doses, cell types, treatment combinations, and experimental conditions, making CPA particularly suitable for pharmacological studies in which response depends strongly on biological context. Subsequent models extended this framework to address specific pharmacological challenges. ChemCPA incorporates molecular structure into perturbation prediction, allowing responses to be inferred for compounds not directly measured in the training data (Hetzel et al., 2022). scVIDR emphasizes dose-dependent responses by integrating latent cellular representations with regression-based prediction (Kana et al., 2023). Together, these approaches improve the generalizability of perturbation models beyond experimentally observed conditions.

CellOT approaches perturbation prediction from a different perspective by modeling how entire cellular populations shift following treatment rather than predicting the response of an average cell. Using neural optimal transport, a method that estimates how one distribution of cell states shifts into another, CellOT learns the transformation between untreated and treated cellular distributions, thereby capturing heterogeneous responses across individual cells (Bunne et al., 2023). More recent models focus on predicting responses to previously unseen perturbations. PRnet predicts treatment-induced transcriptional responses for *in silico* compound screening, whereas PerturbNet estimates post-treatment cellular-state distributions following chemical or genetic perturbations (Qi et al., 2024; Yu et al., 2025). These models are particularly attractive for natural-product discovery because they can prioritize compounds lacking direct perturbation measurements by predicting their likely effects on disease-associated cellular states.

Collectively, perturbation-response models move AI-guided pharmacology beyond predicting molecular interactions toward estimating functional cellular responses. Rather than asking whether a compound binds a target, these approaches ask how a treatment reshapes disease-associated molecular and cellular states. This shift makes perturbation-response prediction particularly valuable for botanical drugs, whose therapeutic effects frequently arise from coordinated modulation of multiple pathways and cell populations rather than a single molecular target. In cardiovascular pharmacology, these models provide an opportunity to predict how botanical drugs modulate disease-associated transcriptional programs across cardiomyocytes, fibroblasts, endothelial cells, and immune cells before experimental validation. These predictions may fail when compounds or cell states fall outside the training domain, and recent benchmarks show that complex models do not consistently outperform appropriate simple baselines (Csendes et al., 2025; Ahlmann-Eltze et al., 2025).

### 4.4 Foundation models and transfer learning

Foundation models represent a recent shift in AI-guided pharmacology by emphasizing large-scale pretraining rather than task-specific model design (Table 1). Instead of learning from a single dataset, these models are first pretrained on large collections of molecular or cellular data to capture general biological patterns and are subsequently fine-tuned for specific pharmacological prediction tasks. In single-cell applications, foundation models are typically pretrained on diverse cell types to learn relationships among genes and cellular states before being adapted to perturbation datasets or disease-specific contexts. This strategy is particularly valuable when labeled data are limited or when predictions must be transferred to previously unobserved biological settings. This adaptation is a form of transfer learning: patterns learned from a large source dataset are reused for a related task with less labeled data.

CRISP illustrates this approach by combining foundation-model representations with transfer learning to predict drug responses in cell types that were not exposed to the same perturbation during training (Wang Y. et al., 2026). The study further demonstrated transfer of drug-response prediction across disease contexts, highlighting the potential of foundation models to improve generalization beyond the original training data. However, their application to cardiovascular ethnopharmacology remains largely conceptual. Whether pretrained models can accurately predict responses to complex botanical preparations or cardiovascular interventions has not yet been systematically evaluated. Natural-product-specific molecular pretraining is illustrated by NaFM, whereas Geneformer represents a single-cell foundation model for learning gene-network relationships from large transcriptomic datasets (Ding et al., 2026; Theodoris et al., 2023).

### 4.5 Integrating AI with network pharmacology

Network pharmacology organizes relationships among botanical metabolites, molecular targets, pathways, and disease phenotypes, whereas AI can help prioritize these relationships by integrating quantitative evidence and estimating context-specific responses. TCM-Agent illustrates an emerging use of large-language-model multi-agent systems to coordinate database retrieval and network analysis (Wang X. et al., 2026). Such systems can improve workflow efficiency, but their outputs remain dependent on database provenance and require source verification and experimental validation. Network analysis can generate hypotheses, but it does not validate them. In a survey of 35 network pharmacology papers, Che and Zhang (2025) found that none reported positive controls for docking. Their benchmark also showed poor agreement between blind docking scores and experimental affinities (Che and Zhang, 2025). Reusing predicted database links to build a network and then presenting that network as support for the same links is circular reasoning. Further docking or molecular dynamics simulations remain computational evidence. Validation of a proposed mechanism requires experiments that test target engagement or activity and whether altering the target changes the response in a disease-relevant model.

### 4.6 Selecting the appropriate AI strategy

The AI approaches described above should not be viewed as competing methods but as complementary tools designed to answer different pharmacological questions (Table 1). Molecular property models estimate physicochemical properties, bioactivity, or toxicity directly from chemical representations. Drug–target models predict interactions between compounds and proteins, whereas perturbation-response models estimate treatment-induced changes in gene expression or cellular state. Foundation models, in contrast, do not define a separate prediction task but provide transferable representations that can improve these prediction tasks when labeled data are scarce. Consequently, the choice of AI strategy should be determined by the biological question, the available data, and the intended stage of drug discovery rather than by algorithmic complexity alone.

## 5 AI-guided discovery frameworks

The computational approaches described above become most powerful when integrated into a discovery workflow. In AI-guided ethnopharmacology, two complementary strategies can be distinguished (Figure 2). The compound-first framework begins with a botanical drug or natural-product metabolite and predicts its biological activities, whereas the disease/target-first framework starts from a disease-associated molecular or cellular state and searches for compounds predicted to reverse it. Although these workflows share many of the same computational tools, they differ in their starting points, prediction objectives, and candidate prioritization strategies.

**FIGURE 2:**
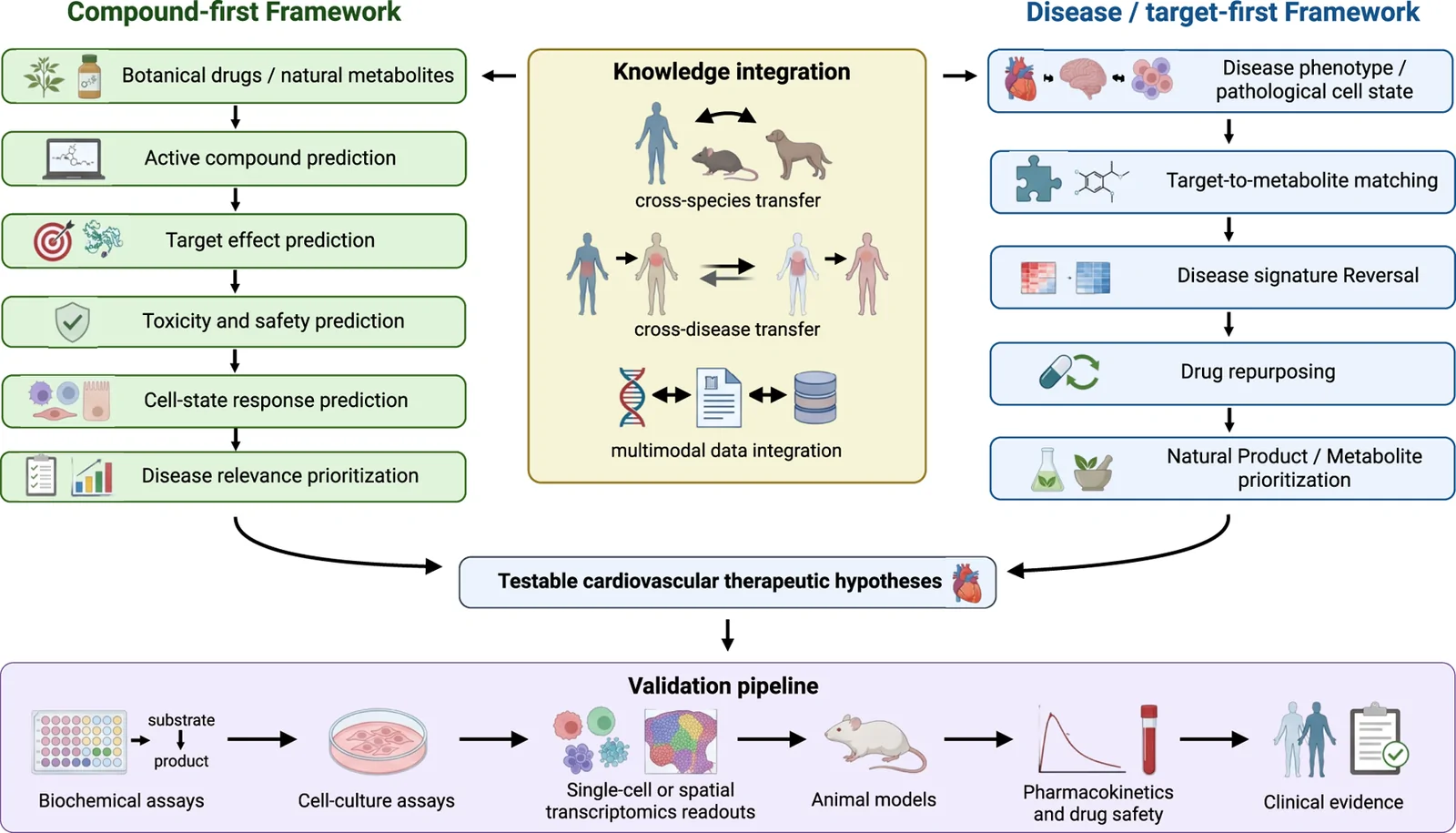
Compound-first and disease-first frameworks for AI-guided ethnopharmacology. The compound-first route asks what a standardized natural product or preparation is predicted to do. The disease-first route asks which candidates may modulate a specified target, phenotype, or pathological cell state. Both frameworks share common biomedical knowledge resources, and the results are subject to experimental validation.

### 5.1 Compound-first discovery

Compound-first discovery begins with a chemically defined botanical drug or natural product metabolite. Purified metabolites can be represented directly by their molecular structures, whereas botanical extracts or botanical formulations require reproducible chemical profiles with quantitative characterization of their major constituents. Ethnomedicinal databases provide a useful starting point for identifying reported metabolites, although analytical validation is essential before these data are used for computational prediction. A complex preparation can be represented initially by submitting its experimentally confirmed metabolites individually to molecular prediction models and interpreting the results together. This constituent-level strategy does not fully capture synergy or antagonism and should be weighted by measured abundance, bioavailability, and achievable exposure before validation in the complete preparation.

For extract-based research, the ConPhyMP guidelines provide specific standards for documenting the identity and processing of the starting material and for reproducible phytochemical characterization (Heinrich et al., 2022). Metabolites proposed to drive an effect should therefore be confirmed in the preparation actually tested, with the source material, extraction process, and batch-level chemical profile reported sufficiently for replication.

Once the constituents have been identified, computational models can estimate properties that influence whether a compound should be studied further. Molecular property models may predict solubility, permeability, toxicity, or related pharmacological features. Drug–target models can suggest molecular interactions for biochemical testing. These predictions provide an initial description of what a compound may be capable of doing, but they do not yet establish how a cell will respond to treatment.

Perturbation-response models provide a more functional layer of prediction. Rather than focusing only on an isolated chemical property or target interaction, these models estimate how treatment may alter gene expression or cellular state. Chemical-structure-aware approaches such as chemCPA and PRnet are particularly relevant when the compound of interest has not been measured in the available perturbation dataset. By representing the compound through its molecular structure, these models can draw information from related chemicals when estimating a transcriptional response (Hetzel et al., 2022; Qi et al., 2024).

The predicted response can then be interpreted in the context of disease. A biologically active compound is not necessarily relevant to the condition being studied. Its effects may also differ across cell populations. A response that appears beneficial in 1 cell type could be ineffective or harmful in another. Cardiovascular cell atlases and disease-associated expression signatures can help determine whether the predicted targets or transcriptional changes occur in relevant cardiac cell populations. The output of a compound-first workflow is therefore not a therapeutic conclusion but a prioritized set of experimentally testable hypotheses. Yoo et al. demonstrated this strategy by integrating heterogeneous pharmacological data to predict medicinal uses for incompletely annotated natural products (Yoo et al., 2020). Their study illustrates how AI can prioritize compounds for further investigation, although prospective experimental validation remains essential.

### 5.2 Disease-first discovery

Disease-first discovery begins with a disease-associated molecular target, signaling pathway, phenotype, or cellular state. If a specific protein has already been implicated, known bioactivity data or drug–target affinity models can be used to identify candidate ligands. When the starting point is a broader molecular phenotype, the search can instead focus on compounds predicted to alter that state.

Transcriptomic signature reversal is a well-established example of this approach. A disease-associated expression pattern is compared with gene-expression changes produced by chemical perturbations. Compounds that oppose the disease signature are then prioritized as possible therapeutic candidates. The Connectivity Map and L1000CDS^2^ provide resources for performing this type of analysis using measured perturbation profiles (Subramanian et al., 2017; Duan et al., 2016). Models such as PRnet extend the same principle by predicting transcriptional responses for compounds that have not been profiled in the specific experimental setting (Qi et al., 2024). Signature reversal is a prioritization signal rather than evidence of therapeutic benefit: similarity metrics may reflect broad control-versus-treatment effects, and any predicted reversal requires disease-relevant functional validation at biologically achievable exposure.

This framework can also support drug repurposing. A compound developed for one indication may produce a molecular or cellular response that is relevant to another disease. CRISP illustrates how foundation-model representations and transfer learning can be used to estimate drug responses in cell types that were not observed under the same perturbation during training (Wang Y. et al., 2026). In principle, a similar strategy could be used to rank natural products against cardiovascular disease states for which direct perturbation data are scarce. Such applications remain prospective and should not be interpreted as evidence that the predicted compound will reproduce the same response in cardiac tissue.

A practical disease-first example would begin with myocardial-infarction cell states defined by human single-cell data, prioritize natural products predicted to normalize the relevant cardiomyocyte, fibroblast, endothelial, or immune programs, and then test the leading candidates in disease-relevant functional assays before animal validation. This sequence is a proposed application of the framework, not a completed end-to-end AI discovery study.

### 5.3 Integrating both workflows

Although conceptually distinct, compound-first and disease-first strategies are highly complementary (Figure 2). A compound-first analysis may identify the response of a drug candidate that is later compared with disease signatures. Conversely, a disease-first search may return a botanical drug whose composition and cellular effects can be further evaluated using the compound-first workflow. In practice, the two routes can form an iterative cycle in which predictions from one direction refine the questions asked in the other (Figure 2).

Ultimately, both workflows pursue the same objective: reducing a vast chemical search space to a manageable set of experimentally testable candidates. Compound-first discovery asks what a known botanical drug is likely to do, whereas disease-first discovery asks which botanical drug is most likely to achieve a desired biological response. Together, these complementary frameworks enable AI to improve candidate prioritization while reducing the cost and complexity of early-stage drug discovery.

## 6 Experimental and clinical validation

Although AI models can achieve strong performance in predicting molecular properties, drug–target interactions, and cellular responses in benchmark settings, computational predictions alone do not constitute pharmacological evidence or therapeutic validation. The strength of evidence in AI-guided pharmacology increases progressively from computational benchmarking to experimental validation and ultimately to prospective clinical evaluation. Accordingly, this section discusses these three complementary levels of validation.

### 6.1 Computational validation

Computational validation is the first step in evaluating AI models for pharmacological prediction. Rather than testing predictions prospectively, most studies assess model performance using experimental measurements that were withheld during training. For molecular prediction, DeepDTA was evaluated against experimentally measured binding affinities from the Davis and KIBA datasets, whereas DeepTox was benchmarked using the Tox21 toxicity challenge (Öztürk et al., 2018; Mayr et al., 2016). Perturbation-response models follow a similar strategy, but the withheld information typically consists of previously unseen perturbations, doses, cell types, or biological contexts.

scGen demonstrated this approach by predicting transcriptional responses in cell types, datasets, and species excluded from model training (Lotfollahi et al., 2019). CellOT similarly evaluated its predictions by comparing inferred and observed distributions of treated single cells using melanoma datasets (Bunne et al., 2023). These studies demonstrate robust out-of-sample prediction but remain examples of computational rather than prospective biological validation.

### 6.2 Experimental validation

Experimental validation extends computational benchmarking by determining whether AI-predicted responses can be reproduced in biological systems. CPA provides an example in which experimentally measured transcriptional responses to withheld drug combinations were accurately reconstructed from single-cell data (Lotfollahi et al., 2023). However, this validation remained at the level of transcriptomic prediction rather than therapeutic efficacy.

Several studies have also evaluated whether AI models can recover established pharmacology. PRnet accurately predicted transcriptional responses and pathway changes for well-characterized compounds, while CRISP prioritized approved therapies and disease-associated pathways consistent with existing experimental and clinical evidence (Qi et al., 2024; Wang Y. et al., 2026). These studies demonstrate that AI models can reproduce known pharmacological relationships, thereby increasing confidence in their predictions. However, recovering established pharmacology does not constitute prospective experimental validation of new therapeutic hypotheses.

Among the studies reviewed here, PRnet provides the strongest example of AI-guided wet-lab validation. Following AI-guided prioritization of large chemical libraries, selected candidate compounds were experimentally tested in human cancer cell lines and showed low-micromolar antiproliferative activity (Qi et al., 2024). These results demonstrate that AI can enrich biologically active candidates for downstream testing. Nevertheless, they do not establish the predicted molecular mechanism or therapeutic efficacy *in vivo*.

For cardiovascular candidates, transcriptional agreement should be followed by functional assays matched to the predicted benefit, such as contractility for heart-failure hypotheses or electrophysiological assessment for arrhythmia risk. When a specific target is predicted, representative target-engagement or causal experiments should distinguish direct mechanism from a correlated phenotypic response. Pharmacokinetic measurements are also needed to determine whether the prioritized metabolite reaches the relevant tissue at a biologically meaningful exposure.

### 6.3 Clinical validation

Current studies provide strong examples of computational validation and a smaller number of cell-based experiments. Several studies also use known drugs, pathways, or structural features as biological controls to support the biological plausibility of model predictions. Despite encouraging computational and cell-based validation, none of the core AI studies reviewed here has advanced an AI-prioritized botanical drug through prospective animal studies and clinical evaluation. Clinical trials of botanical drugs demonstrate how therapeutic efficacy can ultimately be established, but they validate the intervention rather than the AI model that prioritized it. Establishing such an end-to-end validation pipeline will be essential to determine whether AI-guided ethnopharmacology can truly accelerate botanical drug discovery rather than merely recapitulate existing pharmacological knowledge.

Translation of an AI-prioritized botanical drug also requires reproducible identity, manufacturing controls, batch consistency, safety, and clinical evidence. The U.S. Food and Drug Administration botanical-drug guidance illustrates that AI-based prioritization may support development decisions but cannot replace the chemistry, manufacturing, and clinical evidence required for an investigational or marketing application (U.S. Food and Drug Administration, 2016; U.S. Food and Drug Administration, 2025).

## 7 Applications in cardiovascular disease

Ethnopharmacological contributions to cardiovascular therapeutics extend well beyond any single traditional medical system. Historical examples illustrate how traditional and ethnomedicinal knowledge has provided starting points for modern cardiovascular pharmacology. *Digitalis purpurea* L., historically used in European folk medicine and subsequently systematically investigated for the treatment of dropsy, helped establish cardiac glycoside pharmacology, including the development of digitoxin and digoxin as cardiovascular therapeutics (Marullaz, 1991; Whayne, 2018). *Rauvolfia serpentina* (L.) Benth. ex Kurz, used in Ayurvedic medicine, provided reserpine, one of the earliest widely used antihypertensive agents (Jerie, 2007). *Ammi visnaga* (L.) Lam., traditionally used in North African and Middle Eastern medicine, provided khellin, a compound with coronary vasodilatory activity that subsequently served as a chemical starting point in the development of amiodarone (Anrep et al., 1949; Osher et al., 1951; Khalil et al., 2020). These examples illustrate a broader principle underlying AI-guided ethnopharmacology: traditional knowledge can provide biologically informative starting points, whereas contemporary chemical characterization, computational prioritization, and experimental validation are required to translate these observations into modern pharmacological evidence. Although many of the contemporary cardiovascular examples discussed below originate from Traditional Chinese Medicine (TCM), reflecting the availability of recent clinical and computational studies, the computational frameworks described in this review are not restricted to TCM and can be applied to chemically characterized botanical preparations and natural products from Ayurveda, Kampo, Unani, African traditional medicine, Latin American traditions, Indigenous North American medicine, and other ethnomedical systems.

Cardiovascular disease represents a particularly challenging application for AI-guided ethnopharmacology because disease progression involves multiple cell types, dynamic molecular programs, and stage-dependent therapeutic responses. The following examples illustrate how AI can facilitate botanical drug discovery by integrating phytochemical, molecular, cellular, and clinical information across major cardiovascular diseases.

### 7.1 Heart failure, myocardial infarction, and vascular disease

Heart failure is one of the most extensively studied cardiovascular indications for botanical drugs, providing both encouraging clinical evidence and opportunities for AI-guided mechanistic discovery. Reviews of traditional Chinese medicine describe a substantial body of clinical and preclinical cardiovascular research (Hao et al., 2017). The QUEST trial demonstrated that Qiliqiangxin improved clinical outcomes when added to guideline-directed therapy for heart failure with reduced ejection fraction (Cheang et al., 2024). Similarly, a systematic review suggested potential benefits of Shengmai in heart failure, although the quality of the available clinical evidence remains limited, whereas the simplified herbal formula Nuanxinkang has demonstrated beneficial effects in experimental models of cardiac injury and remodeling (Zhou et al., 2014; Chen et al., 2019). These studies demonstrate the therapeutic potential of botanical drugs in cardiovascular disease, yet the bioactive metabolites, molecular targets, and cellular mechanisms underlying these therapeutic effects remain incompletely understood.

Myocardial infarction provides another important example. In the CTS-AMI trial, Tongxinluo improved cardiovascular outcomes in patients with ST-segment elevation myocardial infarction when compared with placebo (Yang et al., 2023). Together, these clinical studies demonstrate that complex botanical formulations can be evaluated using contemporary clinical trial standards. However, the active metabolites and molecular mechanisms responsible for these clinical benefits remain largely unresolved. AI-guided discovery provides a systematic framework for integrating phytochemical composition with molecular and cellular data, thereby prioritizing candidate metabolites and mechanisms for experimental validation.

The botanical composition, plant parts, accepted taxonomic identities, and available standardization information for Qiliqiangxin, Tongxinluo, Shengmai, and Nuanxinkang are shown in Table 2. The completeness of chemical characterization and manufacturing information differs among formulations and studies; therefore, an established formulation name should not be assumed to represent chemically interchangeable products across manufacturers or batches.

**TABLE 2:** Botanical composition and analytical standardization of the cardiovascular formulations.

Formulation	Constituents, accepted latin binomials, and plant parts	Analytical basis
Qiliqiangxin (QUEST trial; Cheang et al., 2024)	1. Astragali Radix—*Astragalus mongholicus* Bunge, root (450 g) 2. Ginseng Radix et Rhizoma—*Panax ginseng* C.A.Mey., root and rhizome (225 g) 3. Aconiti Lateralis Radix Praeparata—*Aconitum carmichaelii* Debeaux, processed lateral root (112.5 g) 4. Salviae Miltiorrhizae Radix et Rhizoma—*Salvia miltiorrhiza* Bunge, root and rhizome (225 g) 5. Descurainiae Semen—*Descurainia sophia* (L.) Webb ex Prantl, ripe seed (150 g) 6. Alismatis Rhizoma—*Alisma orientale* (Sam.) Juz., tuber/rhizome (225 g) 7. Polygonati Odorati Rhizoma—*Polygonatum odoratum* (Mill.) Druce, rhizome (75 g) 8. Cinnamomi Ramulus—*Cinnamomum cassia* (L.) J.Presl, twig (90 g) 9. Carthami Flos—*Carthamus tinctorius* L., flower (90 g) 10. Periplocae Cortex—*Periploca sepium* Bunge, root bark (180 g) 11. Citri Reticulatae Pericarpium—*Citrus reticulata* Blanco, dried mature pericarp (75 g) (amounts are quantities for 1,000 capsules)	Manufactured by Shijiazhuang Yiling Pharmaceutical under Chinese good manufacturing practice requirements. The QUEST report provides a fixed formulation and states that 10 production batches were evaluated using ultra-performance liquid chromatography fingerprints combined with chemometric analysis, with high fingerprint similarity and batch uniformity (Cheang et al., 2024)
Tongxinluo (CTS-AMI trial; Yang et al., 2023)	1. Ginseng Radix et Rhizoma—*Panax ginseng* C.A.Mey., root and rhizome 2. Paeoniae Radix Rubra—*Paeonia lactiflora* Pall., root 3. Ziziphi Spinosae Semen—*Ziziphus jujuba* Mill. Var. *spinosa* (Bunge) Hu ex H.F.Chow, stir-fried mature seed 4. Santali Albi Lignum—*Santalum album* L., stem heartwood 5. Dalbergiae Odoriferae Lignum—*Dalbergia odorifera* T.C.Chen, stem or root heartwood 6. Olibanum—*Boswellia carterii* Birdw. [currently often treated as a synonym of *Boswellia sacra* Flueck.], processed oleo-gum-resin The formulation also contains five animal-derived ingredients—leech, scorpion, ground beetle, centipede, and cicada slough—as well as synthetic borneol	The CTS-AMI trial used manufacturer-controlled 0.26-g capsules and a matching placebo. In another study, the source and collection or processing conditions of the raw materials were controlled, the materials were evaluated according to the Chinese Pharmacopoeia, and high-performance liquid chromatography and gas chromatography fingerprints were obtained (Shen et al., 2014)
Shengmai (studies summarized by Zhou et al., 2014)	1. Ginseng Radix et Rhizoma—*Panax ginseng* C.A.Mey., root and rhizome; red ginseng is used in some Shengmai dosage forms 2. Ophiopogonis Radix—*Ophiopogon japonicus* (Thunb.) Ker Gawl., root tuber 3. Schisandrae Chinensis Fructus—*Schisandra chinensis* (Turcz.) Baill., ripe fruit	A quality-control study applied high-performance liquid chromatography fingerprinting, similarity analysis, principal-component analysis and hierarchical-cluster analysis. It quantified schisandrol A, schisandrol B, methylophiopogonanone A, schisandrin B and the ginsenosides Rf, Rb1, Rg2 and Rb2 as candidate quality markers (Zheng et al., 2020)
Nuanxinkang (Chen et al., 2019)	1. Ginseng Radix et Rhizoma Rubra—*Panax ginseng* C.A.Mey., steamed and dried root and rhizome 2. Ilicis Pubescentis Radix—*Ilex pubescens* Hook. and Arn., root A published experimental study described the two botanical drugs as being combined at a ratio of 23:77 (Dong et al., 2022)	Chen et al. (2019) used ultra-high-performance liquid chromatography–mass spectrometry to identify 21 chemical constituents in Nuanxinkang and incorporated them into network and experimental analyses. Another study reported that concentrated botanical powders were produced under internationally certified good manufacturing practice (Dong et al., 2022)

Single-cell and spatial transcriptomic atlases offer an opportunity to bridge this mechanistic gap by defining disease-associated cellular states at high resolution. Studies of the human heart following myocardial infarction have identified distinct molecular responses in cardiomyocytes, fibroblasts, endothelial cells, vascular smooth muscle cells, and immune populations (Kuppe et al., 2022; Kanemaru et al., 2023). These datasets can define pathological cellular states that serve as targets for disease-first discovery, while perturbation-response models prioritize compounds predicted to reverse these states in specific cell populations. Although this integrated workflow remains largely prospective, it represents one of the most promising applications of AI-guided cardiovascular ethnopharmacology.

The same strategy is particularly attractive for atherosclerosis and vascular disease, where therapeutic responses differ substantially among macrophages, endothelial cells, and vascular smooth muscle cells. Single-cell atlases can identify disease-relevant cellular populations, while perturbation-response models predict how individual cell types respond to treatment. Current studies still rely largely on retrospective network and pathway analyses (Zhai et al., 2025). Future work should directly test AI-predicted responses in disease-relevant cardiovascular cell populations, providing mechanistic validation that complements clinical observations.

Recent studies illustrate applications beyond heart failure and atherosclerosis. A deep-learning model was developed to classify a dyslipidemia pattern used in Chinese-medicine practice, while an integrated machine-learning and network-pharmacology study linked Qishen Yiqi dripping pills with myocardial ischemia–reperfusion injury (Liu et al., 2023; Ge et al., 2026). Machine learning has also been combined with mechanistic experiments for a botanical formula in myocarditis, although clinical validation remains necessary (Zhang et al., 2026). These examples broaden the disease spectrum while also showing that current evidence is heterogeneous and often remains preclinical or retrospective.

### 7.2 Cardiovascular safety assessment

Beyond efficacy prediction, AI may also contribute to cardiovascular safety assessment. Arrhythmia and cardiotoxicity frequently arise from mechanisms that are not captured by conventional toxicity assays. ADMET-AI illustrates how machine learning can predict drug-induced cardiotoxicity from pharmacokinetic and molecular features (Mukherjee et al., 2025). Similar strategies may help prioritize safer natural products before experimental testing. Nevertheless, computational predictions cannot replace electrophysiological, functional, or *in vivo* cardiovascular safety assessment. In cardiovascular patients, candidate botanical drugs must also be assessed for bioavailability, metabolic transformation, and interactions with concomitant therapies such as anticoagulants, antiplatelet agents, and statins; constituent-level predictions cannot establish the safety of the complete preparation.

Collectively, these examples illustrate how AI can complement each stage of cardiovascular botanical drug discovery, from chemical characterization and candidate prioritization to mechanistic investigation and safety assessment. Rather than replacing experimental pharmacology, AI provides a systematic framework for integrating phytochemical, molecular, cellular, and clinical information to generate experimentally testable hypotheses. Ultimately, the value of AI-guided cardiovascular ethnopharmacology will depend not on its ability to reproduce known pharmacology, but on its ability to prospectively identify, experimentally validate, and clinically translate novel botanical therapeutics.

## 8 Challenges and future perspectives

### 8.1 Data quality and standardization

Standards for AI-guided ethnopharmacology should be considered within the Four Pillars of Best Practice, which encompass pharmacological relevance and traditional context, botanical and chemical characterization, basic experimental and ethical rigor, and requirements appropriate to the article type (Heinrich et al., 2020). These standards are particularly important for AI applications because deficiencies in the underlying studies can be propagated into training datasets and computational predictions.

Reliable AI-guided pharmacological prediction depends on accurate representation of botanical materials. Medicinal plants may be recorded under scientific, pharmaceutical, local, or translated names, and a single common name may refer to multiple species. The confusion between *Stephania tetrandra* and *Aristolochia fangchi*, which resulted in aristolochic acid nephropathy, illustrates the clinical consequences of inaccurate botanical annotation (Nortier et al., 2000; Joshi et al., 2008). Such inconsistencies can compromise computational datasets by splitting evidence from the same species or incorrectly merging information from different plants.

Beyond accurate botanical annotation, integrating complementary data types represents another major challenge. Current databases often provide complementary information, but chemical measurements, transcriptomic responses, functional phenotypes, and clinical outcomes are frequently derived from different interventions or experimental settings. HERB 2.0 illustrates the value of integrating clinical and high-throughput evidence within a shared resource (Gao et al., 2025). The next step is to generate matched datasets in which standardized botanical preparations are chemically characterized and evaluated in disease-relevant biological systems. Linking molecular changes to functional phenotypes and exposure measurements will enable AI models to learn relationships that are difficult to infer from unrelated datasets. High-quality, standardized datasets provide the foundation for robust model development, but reliable prediction also depends on rigorous evaluation and interpretation of AI models. Traceable provenance also supports scientific reproducibility. Researchers should retain the original source identifiers, database versions, download dates, and steps used to select and process the data. These records allow others to reconstruct the input dataset and check the evidence underlying the analysis.

### 8.2 Research ethics and responsible data use

AI-guided botanical research should respect the rights of countries over their plant genetic resources and the rights of Indigenous peoples and local communities over associated traditional knowledge. Before using public database records in an AI model, researchers should complete the following checks: (1) Trace the source. Record the plant name, compound identifier, original publication or dataset, and reported country of origin. Identify the source community when traditional knowledge is involved. Record missing information rather than infer the origin from the location of the database; (2) Check the permitted use. Review the database license and any conditions attached to the original records. Use the Access and Benefit-Sharing Clearing-House to identify relevant national requirements and contacts. Confirm whether prior informed consent and mutually agreed benefit-sharing terms are required and whether they cover the planned use. If required permission cannot be confirmed, consult the relevant national authority and knowledge holders before proceeding (Secretariat of the Convention on Biological Diversity, 2011); (3) Check requirements for sequence data. If the analysis uses digital sequence information on genetic resources, consult Target 13 of the Kunming-Montreal Global Biodiversity Framework and relevant CBD decisions, including Decision 16/2. Record the applicable benefit-sharing arrangements and the date of this assessment, as the framework continues to develop (Convention on Biological Diversity, 2022; Convention on Biological Diversity, 2024); (4) Document the decisions. Retain the permission assessment, any required approvals, and agreed benefit-sharing arrangements. Report unresolved limitations and share data and research outputs only as permitted by the applicable conditions.

Researchers using public databases should carefully assess these requirements in relation to the source of the data and its intended research use.

### 8.3 Model robustness and interpretability

Robust model evaluation is essential for determining whether AI models generalize beyond their training data. Compounds that appear different by name may still share highly similar structures, while related cell samples may originate from the same experimental study. If these examples are divided randomly between training and test sets, models may achieve high performance by recognizing familiar patterns rather than learning relationships that transfer to new compounds or biological settings. Joeres and colleagues demonstrated that this type of information leakage can substantially overestimate model performance and developed DataSAIL to generate more realistic data splits (Joeres et al., 2025). Models intended to discover new natural products should therefore be evaluated on structurally distinct compounds, while cardiovascular prediction models should be tested in cardiac or vascular cell types not represented during training.

Model performance should also be interpreted relative to appropriate baseline methods. Recent benchmarking studies demonstrated that simple baseline models can outperform more complex foundation models for post-perturbation transcriptomic prediction under some conditions (Csendes et al., 2025; Ahlmann-Eltze et al., 2025). These findings do not diminish the value of foundation models but emphasize that increasing model complexity does not automatically improve prediction accuracy. Future studies should define the intended application, compare against appropriate baseline methods, and evaluate performance under realistic biological shifts. Evaluation should remain task-specific, using realistic separation of chemical scaffolds, targets, studies, or cell contexts according to the intended application rather than prescribing one universal protocol.

Interpretability represents another essential component of robust AI-guided pharmacology. Feature-attribution methods may identify chemical substructures, genes, or pathways associated with model predictions, but these associations do not establish causal mechanisms (Qadri et al., 2025). PRnet illustrates this principle by experimentally testing AI-prioritized compounds following computational prediction (Qi et al., 2024). Although these studies confirmed biological activity, they did not validate every predicted transcriptional mechanism. Future work should combine computational prediction with targeted mechanistic experiments to establish causal links between AI-generated hypotheses and pharmacological responses.

### 8.4 Prospective validation and future directions

Prospective, end-to-end validation remains the most important challenge for AI-guided ethnopharmacology. None of the AI models discussed in this review has yet advanced an AI-prioritized botanical candidate through the complete discovery pipeline, from computational prioritization and disease-relevant cell models to animal studies and prospective clinical evaluation. PRnet illustrates successful integration of computational prediction with cell-based validation, whereas the QUEST and CTS-AMI trials demonstrate that botanical drugs can be rigorously evaluated in contemporary cardiovascular clinical trials (Qi et al., 2024; Cheang et al., 2024; Yang et al., 2023). However, these studies represent separate stages of the discovery process rather than an end-to-end AI-guided workflow.

Ultimately, the success of AI-guided ethnopharmacology should be judged not by its ability to reproduce existing pharmacological knowledge, but by its ability to prospectively identify, experimentally validate, and clinically translate novel botanical therapeutics. Achieving this goal will require standardized botanical materials, high-quality multimodal datasets, rigorous model evaluation, and prospective biological validation. Future progress will depend less on increasingly complex algorithms than on traceable evidence and carefully designed experiments that directly test AI-generated hypotheses.

## 9 Conclusion

The rapid expansion of chemical, biological, and clinical datasets has transformed opportunities for AI-guided ethnopharmacology. By integrating phytochemical information with multiomics, single-cell, perturbation, and clinical data, AI enables systematic prioritization of candidate metabolites, molecular mechanisms, and disease contexts that would be difficult to identify through conventional approaches alone.

Throughout this review, we have emphasized that AI should be viewed as a hypothesis-generation framework rather than a substitute for pharmacological experimentation. Molecular property prediction, drug–target interaction, perturbation-response modeling, and foundation models each address distinct biological questions and are most powerful when integrated into compound-first or disease-first discovery workflows. Their value lies in improving candidate prioritization while reducing the cost and complexity of early-stage drug discovery.

Despite encouraging progress, AI-guided ethnopharmacology remains limited by data quality, model generalizability, and the lack of prospective validation. Future advances will depend less on increasingly sophisticated algorithms than on standardized botanical materials, high-quality multimodal datasets, rigorous model evaluation, and end-to-end validation from computational prediction to experimental and clinical testing. Ultimately, AI will be judged not by its ability to reproduce existing pharmacological knowledge, but by its ability to prospectively identify, experimentally validate, and clinically translate novel botanical therapeutics.

AI is unlikely to replace pharmacological experiments or clinical trials. Rather, its greatest value lies in accelerating discovery by prioritizing candidate metabolites, linking chemical composition with disease biology, and improving experimental design. When supported by standardized botanical materials, high-quality multimodal data, rigorous biological validation, and prospective clinical evaluation, AI has the potential to complement the empirical and experimental foundations of ethnopharmacology with a more predictive framework for cardiovascular botanical drug discovery.
